# Social connections are differentially related to perceived and physiological age acceleration amongst older adults

**DOI:** 10.1101/2025.02.03.25320261

**Published:** 2025-02-05

**Authors:** Daisy Fancourt, Mikaela Bloomberg, Andrew Steptoe

**Affiliations:** 1 Department of Behavioural Science and Health, UCL, UK

## Abstract

Human social connections are complex ecosystems formed of structural, functional and quality components. Deficits in social connections are associated with adverse age-related health outcomes, but we know little about the ageing-related mechanistic processes underlying this. Using data from 7,047 adults aged 50+ in the English Longitudinal Study of Ageing, we explored associations between diverse aspects of social deficits and both perceived and physiological age acceleration, which provide complementary psycho-behavioural and biological mechanistic explanations. We created and validated a novel physiological ageing index using clinical indicators pertaining to the cardiovascular, respiratory, haematologic, metaboloic and cognitive systems using principal component analysis. Doubly-robust estimations using inverse-probability-weighted regression adjustment estimators showed that living alone, low social integration and high social isolation were risk factors for physiological age acceleration, with those who lived alone on average 1.9 years older than those who lived with others (95% CI 0.9–3.0 years older; 32% greater age acceleration than people who live with others). However, social deficits were not related to accelerations in perceived age. Analyses were robust to multiple sensitivity analyses and maintained four years later. These findings provide important mechanistic insight that helps to explain the relationship between social deficits and age-related morbitidy and mortality outcomes.

## Introduction

Human social connections are complex ecosystems formed of structural components (such as the size of social networks and frequency of social contact), functional components (including the social and emotional support provided by these networks and contacts) and quality components (including the positive and negative experiences our interactions bring).^1^ Deficits in these social connections (“social deficits”) like loneliness and social isolation are detrimental to health across the life-course, but they pose a particular challenge in older age. Multi-national estimates of the relationship between loneliness and age show U-shaped curves, with loneliness being higher amongst younger people but also steadily increasing from around age 60 onwards ^2,3^. Similarly, social isolation becomes more prominent as people age, with severe social isolation being 4 times more likely in those aged >90 compared to aged 65–69.^4^ However, while we know that social deficits increase with age, we know comparatively little about the reverse: how social deficits affect processes of ageing.

Social deficits are clearly and strongly associated with adverse health outcomes, including the incidence of physical diseases (e.g. cardiovascular disease/dementia/diabetes),^5–8^ psychiatric disorders (e.g. depression/anxiety/ schizophrenia),^9,10^ age-related decline,^11,12^ and mortality (through suicide and other causes).^13–15^ Many of these are outcomes that increase in prevalence as age advances. Recent biological research has identified a number of mechanistic pathways that link social deficits to these age-related outcomes, including (i) an overactive HPA axis resulting in receptor cells developing glucocorticoid resistance, leading to a greater susceptibility to inflammation, (ii) dysregulation of the autonomic nervous system’s ability to regular cardiovascular activity, (iii) maladaptive changes in immunological responses, (iv) reduced repair and restorative processes, and (v) increased brain atrophy and reduced neurogenesis.^16–18^ These biological processes are notable because they themselves include important hallmarks of ageing, which do not necessarily advance in tandem with chronological age.^19^ Over the past decade, there has been increasing interest in the use of biological clocks that combine age-related molecular features to identify discrepancies between chronological vs biological age (i.e. accelerated vs decelerated ageing). The first ageing “clocks” were initially developed using DNA methylation data, and there is already promising initial data suggesting that deficits in social connections are related to accelerated epigenetic ageing.^20,21^

But other clocks based on more downstream molecular phenotypes like proteomic and metabolomic data and broader clinical data are increasingly being developed and function well in predicting age-related pathology.^22^ One type of clock increasing in popularity is a physiological clock that combines phenotypic measures of blood-based biomarkers (e.g. fibrinogen, C-reactive protein and glycated haemoglobin) with tests of physiological function (e.g. grip strength, respiratory function, pulse pressure and blood pressure) ^23–25^. Acceleration of physiological age, as measured by this type of clock, relative to chronological age has been shown to predict multiple age-related outcomes including cardiovascular disease, arthritis, osteoporosis, cognitive dysfunction, dementia and mortality ^23–25^. Significantly, the individual components of this physiological ageing index have previously been shown to be influenced by social deficits.^26–29^ So, we could hypothesise that social deficits therefore influence age-related morbidity and mortality through reducing overall physiological age.

In addition to increasing interest in objective biological ageing, there is also growing awareness about the importance of differentiating chronological age from subjective or “perceived” age. Perceived age (asking an individual how old they feel) is imbued with cultural, social and personal meaning, including reflecting social norms and personal desires. But it can provide important insight into aspects of health and physical function alongside feelings of vitality, wellbeing, optimism and self-esteem.^30,31^ These are all psychological processes that themselves are related to lower risk of age-related morbidity and mortality.^32^ Additionally, perceived age provides an assessment of a person’s sense of their own capacity that they may find it harder to articulate or that may be harder to fully capture in quantitative surveys. Notably, perceived age can be an important biomarker in its own right: people who feel younger than they are chronologically have lower mortality risk, even after controlling for existing health status.^30,33^ As such, perceived age may provide a mental map of one’s remaining life expectancy, providing complementary insight into ageing to more objective biological ageing clocks. But whereas there is a strong literature on how perceived age relates to age-related morbidity and mortality, there is much less research into factors associated with a lower perceived age or perceived age deceleration.

Consequently, perceived and physiological age provide different but complementary insight into mechanistic processes that could link social deficits to age-related morbidity and mortality. Therefore, this paper aimed to explore how deficits in our social connections are related to decelerations or accelerations in perceived and physiological age. We used a theoretically-informed model of social connections that takes account of their structural, functional and quality factors and made use of a representative cohort of older adults living in England.^26^ Our research questions were (i) are strong social connections related to decelerated ageing, either perceived or physiological? (ii) how do potential effects differ based on structural, functional and quality aspects of social relationships? And (ii) is there synergy in the relationship between social connections and rates of perceived and physiological ageing?

## Methods

### Dataset

Data were derived from the English Longitudinal Study of Ageing (ELSA). ELSA is a large-scale panel study of people aged 50 and over and their partners, living in private households in England. The original sample was drawn from participants from the Health Survey in England (HSE) in 1998, 1999 and 2001.^34^ The first wave of data collection commenced in 2002/2003, and participants have been followed biennially since. We used wave 2 (2004/5), as this wave included a rich battery of social and physiological variables of relevance.

We restricted participants to core ELSA members who had returned the self-completion questionnaire where most of our main variables of interest were measured (n=8,354), who were over the age of 50 (n=8,129), and who had full data on exposures and confounders (n=7,047). Within this sample, 6,621 had data on perceived ageing, and 4,169 had full data on physiological ageing. In main analyses, the final sample was allowed to vary between analyses for perceived and physiological age to maximise power, but sensitivity analyses restricted the sample just to individuals who had both perceived and physiological ageing (n=3,928).

### Exposures

#### Structural

Structural aspects of social connections included living alone (vs with others), intimate network size (how many children, family and friends respondents had close relationships with; count variable split into lowest quartile indicating most deficit vs other quartiles), social integration (number of organisations, clubs and societies respondents were a member of, their engagement with diverse volunteering activities, and their engagement with diverse cultural activities; count variable split into lowest quartile of integration vs other quartiles), and social isolation (whether respondents saw or spoke on the phone to family, friends or children less than once a week, resulting in a scale for each from 0–6, with the scores averaged and split into most isolated quartile vs other quartiles).

#### Functional

Functional aspects of social connections included loneliness (measured using the three-item subscale from the revised UCLA loneliness scale, resulting in a scale from 3–9 split into most lonely quartile vs other quartiles [22]), and perceived social support (whether participants feel they can rely on others, can open up to them, and are not let down by them, asked separately for partner, children, relatives and friends with each question scored from 1–4, with the scores averaged and split into lowest social support quartile vs other quartiles).

#### Quality

Quality aspects of social connections included social strain (whether participants feel they are criticised by others, not understood by them, and they get on their nerves, asked separately for partner, children, relatives and friends with each question scored from 1–4 generating a scale of 12–48 split into highest strain quartile vs other quartiles).

**Figure 1:**
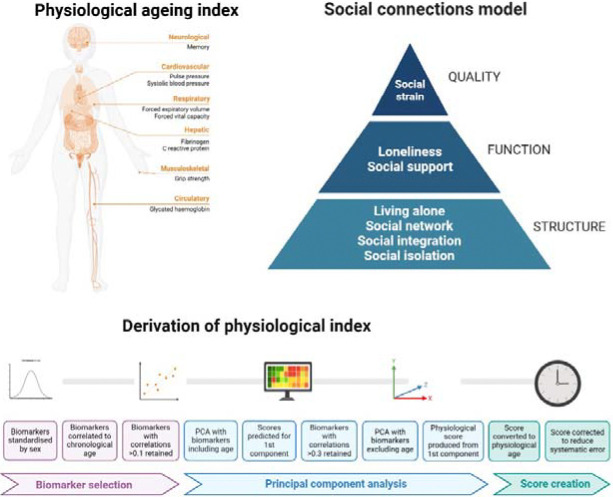
Models of social connections and physiological ageing

Social connections measures are summarised in Figure 1 and full details of questions are provided in Supplementary Table 1.

### Outcomes

A **perceived age acceleration** measure was derived by asking participants how old they felt (measured in years). To create the perceived age acceleration/deceleration measure, chronological age was subtracted from perceived age. Whereby negative scores indicate decelerated ageing (slower perceived ageing), and positive scores indicate accelerated ageing (faster perceived ageing).

A **physiological age acceleration** measure (measured in years) was derived using the principal component analyses (PCA) method, an established method to measure physiological ageing (Jia et al., 2017; Nakamura et al., 1988). It was created using clinical indicators pertaining to the cardiovascular system (pulse pressure, systolic blood pressure, diastolic blood pressure, mean arterial pressure), respiratory system (forced vital capacity [FVC], forced expiratory volume in one second [FEV]), the haematologic system (haemoglobin concentration, fibrinogen, C-reactive protein [CRP], ferritin), metabolic system (fasting glucose, glycated haemoglobin, total cholesterol, LDL cholesterol, HDL cholesterol, triglyceride), muscle and fat (grip strength, waist circumference), and cognitive system (memory tests of immediate and delayed recall). All measures were taken at wave 2 in nurse visits (blood -based biomarkers and physiological testing) or at the main ELSA interview (cognitive testing). This index followed the process previously described for creating a physiological ageing index,^25^ but with the addition of memory as a cognitive component. The full details of physiological age derivation using the PCA method are summarised in Figure 1 and Supplementary Materials (Appendix 1). To create the physiological age acceleration/deceleration measure, chronological age was subtracted from physiological age. Negative scores indicate decelerated ageing (slower physiological ageing), and positive scores indicate accelerated ageing (faster physiological ageing). Analyses of this index show its association with incident functional and mobility limitations, memory impairment, and diverse chronic conditions in the ELSA study population (Supplemental table 2).

### Confounders

Factors identified as predicting both receptive arts engagement and mortality were identified using directed acyclic graphs (DAGs) and included as covariates ^35^. Demographic and socio-economic confounders included chronological age (in years), gender (male or female), ethnicity (white British vs other), educational qualifications (no educational qualifications; education to GCE/O-levels/national vocational qualification (NVQ) 2 (qualifications at age 16); education to NVQ3/GCE/A-levels (qualifications at age 18); higher qualification/NVQ4/NVQ5/degree), total non-pension wealth (which combines net financial and physical wealth plus net owner-occupied housing wealth; categorised in quintiles) ^36^, and house ownership (whether individuals owned their property outright vs with a mortgage/renting/social housing/other).

Health and behavioural confounders included whether participants were sedentary (categorised as engaging in mild, moderate or vigorous activity less than once a week), frequency of alcohol consumption (more than 5 days a week vs less), whether participants currently smoked, and whether participants reported currently having a diagnosis of any chronic conditions including cancer, lung disease or cardiovascular disease (including high blood pressure, angina, a previous heart attack, heart failure, a heart murmur, an abnormal rhythm, diabetes, a previous stroke, high cholesterol, or other heart trouble), arthritis, asthma, osteoporosis, Parkinson’s disease, Alzheimer’s disease or dementia. We additionally looked at the number of difficulties in carrying out activities of daily living that participants reported (ADLs; including dressing, bathing, eating, using a toilet, shopping, taking medications or making telephone calls), self-reported health (poor, fair, good, very good, excellent), BMI (<25, 25–30, ≥30), and depression (using the Centre for Epidemiological Studies Depression (CES-D) scale, ≥3).

### Statistics

Data were analysed using doubly robust estimation using the inverse-probability-weighted regression adjustment (IPWRA) estimator. This involves building two models to account for the non-random treatment assignment: a regression adjustment model for the outcome and a treatment-assignment model for the exposure, only one of which has to be correctly specified, enhancing the robustness of the analysis.^37^ This approach thus provides two chances to make valid inferences, instead of just one, as long as either the treatment group or outcome can be determined as a function of the covariates and there are no unobserved confounders. IPWRA estimators apply weighted regression coefficients to compute averages of treatment-level predicted outcomes, where the weights are the estimated inverse probabilities of treatment. Following our DAGs, confounders applied to both exposure and outcome were sex, ethnicity, wealth, education, house ownership, sedentary behaviours, alcohol consumption, smoking, and chronic conditions. Problems with ADLs and self-reported health were additionally applied to the exposure, and BMI and depression to the outcome.

We estimated the average treatment effect in the population (ATE), which is the average difference in outcome if everyone in the population experienced the exposure, versus no-one in the population. We also estimated the potential-outcome means (POMs) by regression adjustment, which use contrasts of averages of treatment-specific predicted outcomes to estimate treatment effects, and used this to calculate the ATE as a percentage of the untreated POM (presented with the delta-method-based standard error).^38^ Separate models were estimated for each of the eight exposures, so a Bonferroni alpha of 0.05/8 or 0.006 accounts for multiple corrections. For all analyses, we applied probability weights for the self-completion questionnaire provided in the data, which account for complex sampling strategies and non-response.

Depression could plausibly lie on the causal pathway from social connections to ageing, so it was only included in the outcome confounder model, not the exposure confounder model, in the main analyses. However, a sensitivity analysis additionally accounted for depression within the exposure model. A second sensitivity analysis restricted the sample to only people who had data both for the perceived and physiological ageing indices. A third sensitivity analysis excluded anybody whose perceived or physiological age was more than 30 years above or below their chronological age to assess the stability of the findings without the influence of outliers. A fourth sensitivity analyses dichotomised the results by age (split at 65 years) in order to ascertain whether results were consistent across ageing. A fifth sensitivity analysis recategorized the quartiles such that the quartile showing least sign of social deficit and most sign of strong social connections was the “exposed” population to ascertain whether the findings were the same for strong rather than weak social connections. A final sensitivity analysis repeated the main analyses but using perceived and physiological age acceleration measured four years later, to assess whether findings held longitudinally as well as cross-sectionally.

## Results

### Descriptive statistics

Of the 7,047 adults included in the analyses, the average age was 65.6 years (SD 9.4) and 55.2% were female.

**Figure 2:**
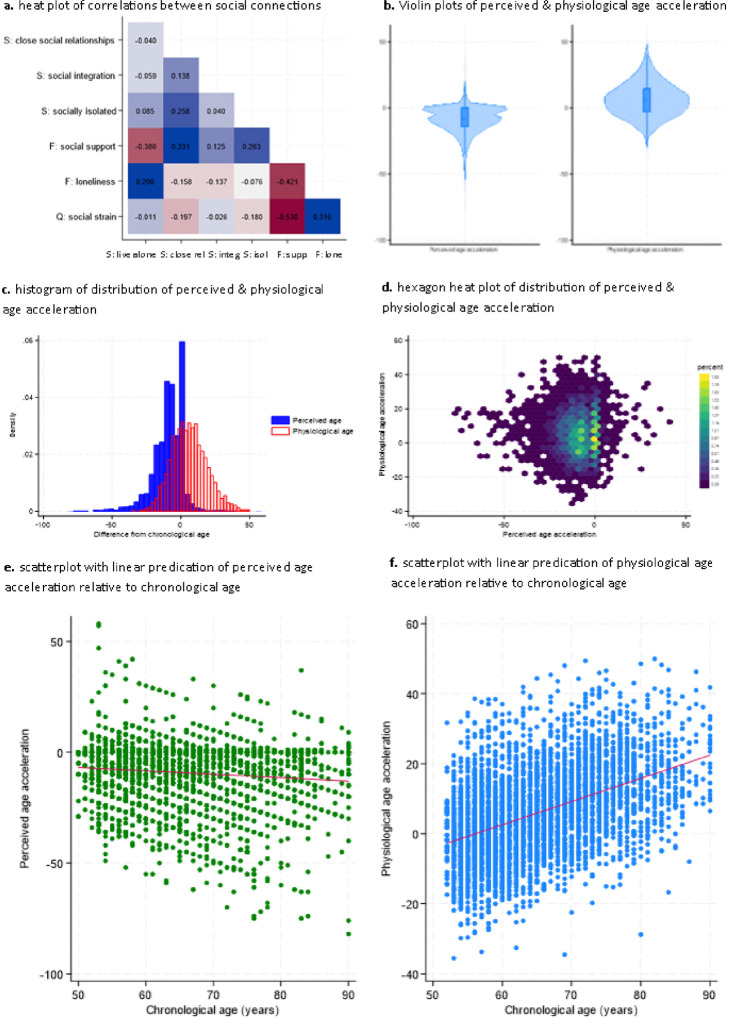
Descriptive figures of exposure and outcome

There were only weak to moderate correlations between measures of social deficits, supporting their inclusion as separate items within the analyses (figure 2a), although people who had clear deficits in structural social factors like living alone scored lower on functional aspects like having lower social support and higher loneliness, and the quality of social relationships was generally poorer amongst those with poorer functional aspects too.

Notably, there was no correlation between perceived and physiological age (r=−0.01, p=.56) (histogram and heatmap shown in figures 2c and 2d). People almost exclusively rated their perceived age to be the same or lower than their chronological age (92% the same or below), while physiological age was more evenly distributed above and below chronological age, with a slight skew towards people being older physiologically than chronologically (33% the same or below, figure 2b). There was a slight correlation between older chronological age and lower perceived age and a marked correlation between older chronological age and greater physiological age acceleration (scatterplots in figures 2e and 2f and hexagon heatmaps in supplementary figures 1a and 1b).

### Main analyses

#### Structure

**Figure 3:**
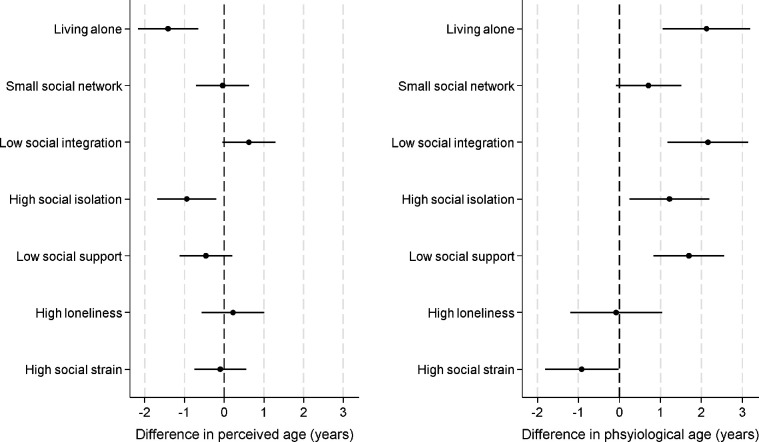
relationship between social deficits and perceived and physiological age acceleration

People who lived alone perceived their age to be 1.6 years younger than it was (95% CI 0.8 to 2.4 years younger), which was 18% lower than people who live with others (for full results see Figure 3 & Supplementary Table 3). But their physiological age was 1.9 years older (95% CI 0.9 to 3.0 years older; 32% greater age acceleration than people who live with others). Similarly, people who were most socially isolated perceived themselves be 1 year younger than their chronological age (95% CI 0.2 to 1.7 years younger; 10% greater age deceleration than those not socially isolated), whereas their physiological age was 1.2 years older (95% CI 0.2 to 2.2 years older; 19% greater age acceleration than those not socially isolated). There was no association between having a small social network and perceived or physiological age acceleration. People who had low levels of social integration also did not have perceived age acceleration, although they did show patterns of accelerated physiological age (2.1 years older, 95% CI 1.1 to 3.1 years older, 36% greater age acceleration than those who were more socially integrated).

#### Function

People who had low levels of social support or high levels of loneliness showed no differences in the perceived age acceleration. But low social support was associated with physiological age acceleration (average 1.6 years older, 95%CI 0.7 to 2.5 years older, 26% greater age acceleration than those without low social support).

#### Quality

High social strain was not related to perceived or physiological age acceleration, but it did show some marginal associations with physiological age deceleration (1.6 years younger, 95%CI −1.75 to 0.01, p=.051).

### Sensitivity analyses

Overall, results were largely consistent across sensitivity analyses, although the physiological age deceleration associated with high social strain was attenuated when taking account of depression and outliers (Supplementary Figures 2–4). Psychological age deceleration results were strongest for living alone in adults under the age of 65 and for social isolation for adults over the age of 65 (Supplementary Figure 5). Notably, adults under the age of 65 did report feeling older if they had low levels of social integration. For physiological age acceleration, low social integration and low social support were clearest predictors in under 65s, whereas all results from the main analyses were evident in over 65s.

**Figure 4:**
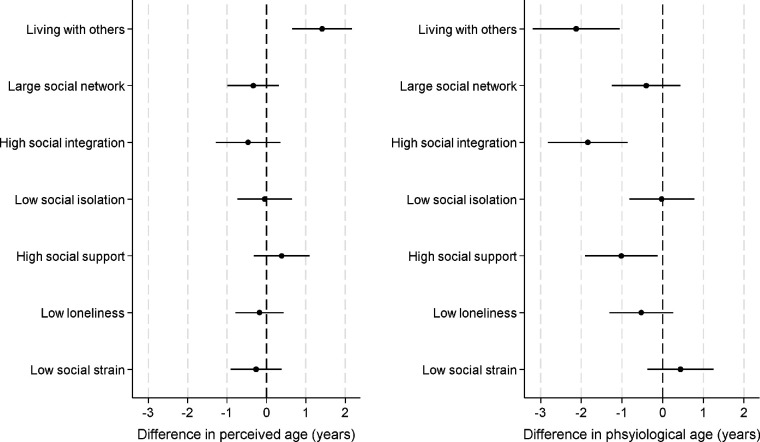
relationship between positive social connections and perceived and physiological age acceleration

When considering whether strong social connections might be protective, living with others vs living alone showed the same findings as for social deficit analyses (just in reverse) given the binary nature of the variable (Figure 4). No other factors were associated with perceived age deceleration, but high social integration and high social support but were both associated with physiological age deceleration. Those with high social integration were physiologically 1.8 years younger (95%CI 0.8 to 2.8 years younger, 26% greater age deceleration than those without high social integration). Those with high social support were physiologically 0.9 years younger (95%CI 0.04 to 1.8 years younger, 14% greater age deceleration than those without high social integration). Finally, when using perceived and physiological age acceleration measures from four years later, results were largely maintained, with the exception of associations for social isolation with both perceived and physiological age acceleration, although coefficients were smaller.

## Discussion

Our findings show that our patterns of social connections are related in highly nuanced ways to perceived and physiological patterns of age acceleration. Deficits in structural aspects of social connections showed the strongest associations with objective measures of physiological age acceleration, with living alone, low social integration and high social isolation being risk factors for age acceleration. However, this was markedly at odds with people’s perceptions of their ageing. No aspects of social connections were related to accelerated perceived age, which was perhaps unsurprising given only 8% of people perceived themselves to be older than they were. Two social factors were related to slower perceived age somewhat counterintuitively: those who live alone and are highly socially isolated perceived themselves to be younger. Similarly, there was no association between the functional and quality aspects of people’s social connections and their perceived ageing, but low social support was associated with accelerated physiological ageing. There was also no evidence that the number of friends was related to perceived or physiological age acceleration. These associations were most prominent in adults as they became older. In reverse, living with others, high social integration and high social support were associated with slower physiological ageing.

The relationship between social deficits and accelerated physiological ageing echoes previous research showing adverse physiological effects of poor social connections, including for individual components of the physiological ageing index we used, such as cardiovascular risk factors, cognitive function, and inflammatory biomarkers.^26–29^ However, by demonstrating a relationship between social deficits and the combined index of these phenotypic markers that considers their predictive potential for ageing, we provide novel insight into a plausible mechanism by which social deficits could influence age-related morbidity and mortality. It is notable that structural factors had the strongest association with physiological ageing. In considerations of the relative risk of deficits to structural, functional and quality aspects of social connections, deficits to structure have been proposed as the most damaging, partly because structural factors underlie the capacity for an individual to experience good function or quality of relationships.^26,39^ However, when considering which factors could be risk-reducing, only a subset of those factors whose deficits were adversely related to physiological age acceleration appeared protective when experienced in high quantities. Living with somebody was associated with the greatest age deceleration, but social integration was similarly protective. This could be because social integration involves diverse community and leisure activities that not only provide social connections but also bring additional salutogenic ingredients such as physical activity, cognitive stimulation, opportunities for multiple social identities, and often creativity and imagination, all of which are related to better health over time.^40,41^ That said, it is important to note that these findings are likely bidirectional, as for many mechanisms relating social behaviours with health, with physiological functioning potentially not only influenced by social deficits but also affecting one’s ability to engage in social behaviours. Notably, when we used outcome measures from four years later, results were largely maintained, although coeffieicents were smaller. This attenuation was not very prominent for living alone but more the case for social integration and social isolation, where poor health can create barriers to social behaviours.

An important consideration is why there were such different results for perceived vs physiological ageing. Notably, there was almost no correlation between the two types of age acceleration, with just 0.01% of the variance in one explained by the other. Only 8% of people perceived themselves to be older than their chronological age, compared to 67% being physiologically older on the index we used, which echoes similar distinctions between perceived and biological age found in other studies.^30^ In some instances, such as for living alone and high social isolation, there was a direct mismatch between perceived and physiological findings. It is possible that compensatory effects were at play here, with individuals who live alone or who are more isolated having a lower personal need for social contact (e.g. due to personality type) or justifying these social deficits internally through perceiving themselves to be better off without more social connections. This compensatory effect could plausibly be reported for isolation and living alone but not other aspects of social connections as these are some of the most self-evident objective manifestations of social deficits. It could also be that those who live alone have to stay more functionally independent to manage their own homes, and that those who are more socially isolated have less opportunity to experience age-related discrimination, which is an experience related to perceived age.^42^ However, such theories remain to be tested further through alternative research methods.

Overall, these findings are important as they provide insight into how social deficits influence age-related pathology. The stronger relationship with physiological ageing suggests that objective biological and functional processes may be more important mechanistically to the adverse health effects of social deficits than broader processes related to subjective perceptions. The strength of the relationship with physiological ageing appeared to get stronger with chronological age, suggesting both that the importance of social connections – and the importance of providing interventions to increase these connections - may increase over time, and potentially that increasing physiological age also bidirectionally has a greater influence on social behaviours as people become chronologically older.

Our study has many strengths including drawing its data from a large, representative sample of older adults, its theoretically-informed approach to exploring social connections, its use of a multi-dimensional model of physiological age that has been independently associated with diverse aspects of age-related pathology, its parallel consideration of subjective and objective processes in ageing, and its statistical use of doubly-robust estimators, which provide a clear advance on more traditional regression-based approaches to conditioning on counterfactuals. However, there are some limitations. We were limited by the measures of social connections included in the datasets. So we only had data on negative aspects of social quality, not positive ones. As this was the first study into complex patterns of social connections and age acceleration, we focused on identifying a cross-sectional ‘signature’, albeit with some explorations of the stability of this over the following four years. But future studies are encouraged to assess whether social deficits affect rate of perceived and physiological age acceleration. Finally, while we included identified confounders and used a statistical method that allows two different model specifications for exposure and outcome (only one of which has to be correct), residual confounding remains possible.

In conclusion, there is no evidence that having friends helps older adults to stay younger either in terms of their perceived or physiological age. But lack of contact with friends and other family and relatives is associated with faster physiological ageing, even if we perceive the contrary ourselves. These findings provide important mechanistic insight that helps to explain the relationship between social deficits and age-related morbitidy and mortality outcomes. Future studies are encouraged to test whether building the structural foundations for good social connections may be a risk-reducing strategy for age acceleration. Given the discrepancy between people’s perceived and physiological ageing, individuals may be unaware of the underlying deficits and benefits of social connections, so enhancing awareness of the impact that social connections have on our health remains an important public health strategy.

## Supplementary Material

Supplement 1

## Figures and Tables

**Table 1:** Sample descriptives

	Mean (SD) / Proportion (%)

Chronological age (years)	65.6 (9.3)
Perceived age (years)	56.1 (13.4)
Physiological age (years)	71.4 (18.6)
Sex	
Male	3,155 (44.8%)
Female	3,892 (55.2%)
Ethnicity	
White	6,951 (98.6%)
Not White	96 (1.4%)
Working status	
Not working	4,566 (64.8%)
Working full/part-time	2,481 (35.2%)
Housing tenure	
Other	2,574 (36.5%)
Outright homeowner	4,473 (63.5%)
Physically inactive	
no	6,648 (94.3%)
yes	399 (5.7%)
Alcohol consumption	
<5 times a week	5,162 (73.3%)
5+ times a week	1,885 (26.7%)
Current smoker	
no	6,051 (85.9%)
yes	996 (14.1%)
No. of chronic conditions	
no	5,986 (84.9%)
yes	1,061 (15.1%)
Depression (CESD>=3)	
no	5,588 (79.3%)
yes	1,459 (20.7%)
Net non-pension wealth (quintiles)	
1 - lowest wealth quintile	1,305 (18.5%)
2	1,385 (19.7%)
3	1,430 (20.3%)
4	1,457 (20.7%)
5 - highest wealth quintile	1,470 (20.9%)
Educational attainment	
Degree	919 (13.0%)
nvq3 A level/higher education	2,016 (28.6%)
nvq2/gce o level	4,112 (58.4%)
No. of ADL limitations	
0	5,795 (82.2%)
1	698 (9.9%)
2+	554 (7.9%)
Self-reported health	
Poor	428 (6.1%)
Fair	1,316 (18.7%)
Good	2,269 (32.2%)
Very good	2,092 (29.7%)
Excellent	942 (13.4%)
BMI	
BMI<25	1,949 (27.7%)
BMI 25–30	3,054 (43.3%)
BMI>=30	2,044 (29.0%)
